# Frailty index in the Colonias of the Rio Grande Valley: health related quality of life and resilience

**DOI:** 10.3389/fmed.2023.1240494

**Published:** 2023-11-27

**Authors:** Eron G. Manusov, Vincent P. Diego, Sarah Williams-Blangero

**Affiliations:** ^1^Department of Human Genetics, School of Medicine, University of Texas Rio Grande Valley, Brownsville, TX, United States; ^2^South Texas Diabetes and Obesity Institute, School of Medicine, University of Texas Rio Grande Valley, Brownsville, TX, United States

**Keywords:** frailty, Mexican American, health-related quality of life, resilience, South Texas, Frailty Index

## Abstract

**Background:**

Frailty is characterized by an accumulation of deficits that lead to vulnerability to adverse health outcomes. The Frailty Index (FI) quantifies frailty by measuring deficits that increase susceptibility to stressors. This study focused on a population of Mexican Americans living in vulnerable communities in the Rio Grande Valley of south Texas. We used a Frailty Index developed based on common health-related data--the Patient Health Questionnaire (PHQ-9) and a Health-related Quality of Life survey (Duke Health Profile). Quality of life, resilience, and frailty are interrelated and influenced by chronic illness, mental illness, poverty, cognitive impairment, and community support.

**Methods:**

We used Logistic regression analysis, factor component analysis, receiver operating characteristic curves, and odds ratios to identify potential associations between clinical variables and candidate predictor variables and seven physiological health variables, and two survey instruments. We analyzed data obtained from participants (894) that live in two Colonias located on the Texas-Mexico border. We calculated the FI with seven physiological variables, PHQ-9 score, and the 11 domain-specific Duke Profile scores, for a total of 19 health deficits. We then dichotomized FI (>0.25) and determined ROC curves through model selection to determine best predictors of frailty.

**Results:**

Females (*n* = 622) had a higher starting frailty, and males (*n* = 272) had a significantly greater change rate with age. Women score higher in anxiety, depression, anxiety/depression, and pain. The frailty index and quality of life markers are strongly inversely related; poorer quality of life leads to greater frailty independent physiological health variables, the PHQ 9, sex, and age.

**Conclusion:**

The study highlights the importance of addressing modifiable mental health and social stressors to reduce frailty. Furthermore, it suggests that factors supporting resilience and well-being, such as physical and mental health, social support, and perceived health, play a crucial role in frailty development. The findings have implications for interventions targeting vulnerable populations and emphasize the need for further research on the relationship between health-related quality of life and frailty.

## Introduction

Frailty is associated with psychosocial and biological mechanisms, including quality of life, physical decline, and overwhelmed resilience related to stressors (1–4). Frailty is a phenotype and is an accumulation of deficits characterized by vulnerability to adverse health outcomes (5, 6). The Frailty Index (FI) measures deficits that increase vulnerability to stressors and quantifies the level of frailty, focusing on the number and nature of deficits contributing to frailty (7, 8). The Frailty Index (FI) may incorporate social, physical, and psychological contributors and Frailty Index are consistently associated with frailty risk regardless of the specific factors measured (9–11). We calculate a FI as the number of deficits/total number of the deficits. Using data from Mexican American participants living in vulnerable communities (Rio Grande Valley Colonias) who sought healthcare from a mobile medical unit (UniMóvil), we developed a Frailty Index using 19 commonly measured health-related data (obesity hypertension, high triglycerides, low high-density lipoprotein, high low-density lipoprotein, high cholesterol), the Patient Health Questionnaire (9 domains), and a Health-related Quality of Life survey (Duke Health Profile) (1). We chose the 17-item self-report questionnaire HrQoL Duke Health Profile (reliability 0.30–0.78) (12) because of the inclusion of six health-function measures (physical, mental, social, general, perceived health, and self-esteem) and four health-dysfunction measures (anxiety, depression, pain, and disability). The Patient Health Questionnaire (PHQ-9; reliability 0.89, sensitivity 88%, and specificity 88%) is a commonly used screen for depression (13). We used the 19 measures because of the high prevalence in the Rio Grande Valley (RGV) of obesity (55.5%), hypertension (39%), diabetes (32.5%), and depression (19%) (14, 15). Initial data analysis supported our population’s high frailty prevalence, mirroring chronic illness’s prevalence. We found a higher prevalence of frailty in the older, more established Colonia (Cameron Park) within Brownsville, Texas, and in younger women. Frailty peaked at 40–60 years, and we found that although women had a higher FI earlier in life, men became more frail with age (most likely due to increased chronic illness and hard physical labor) (1).

HrQoL, resilience, and frailty are intimately interconnected (9, 16). Chronic illness, sarcopenia, mental illness, poverty, cognitive impairment, lower psychological well-being, and the degree of community support, affect HrQoL (17–24). Excessive treatment of chronic illness may also affect HrQoL and frailty (23). A study of community-dwelling older adults demonstrated statistically significant negative associations between physical frailty, psychological frailty, and all dimensions of HrQoL (25). Conversely, resilience is associated with protection from frailty (2, 4, 26–31).

We are conducting this study as an extension of our previous research, which highlighted the utility of a FI incorporating standard clinic and HrQoL measures in the healthcare of Mexican Americans in South Texas. Our current investigation aims to assess the influence of HrQoL and resilience on frailty among Mexican Americans facing vulnerable and at-risk living conditions (1). Our primary objective is to identify the specific measures that predict frailty in this population. Additionally, we aim to explore whether physical and psychological functions, as well as dysfunctions, serve as predictors of frailty. By analyzing the deficits measured, we can establish a reciprocal relationship between these factors, shedding light on their interplay with resilience, health-related quality of life, and frailty.

## Materials and methods

The University of Texas Rio Grande Valley Institutional Review Board approved the protocol. All patients signed consent for care. The study adhered to the ethical guidelines of the Declaration of Helsinki. The datasets presented in this study can be found in online repositories. The names of the repository/repositories and accession number(s) can be found at: Manusov (32).

Health-related quality-of-life questionnaires and depression surveys are routinely administered as part of the care provided by the mobile medical van clinic (UniMóvil) as part of an effort to address the community’s medical, mental health, and social needs. The clinical team travels to the Colonias bi-weekly and collects information such as body mass index, blood pressure, glycated hemoglobin, lipid levels, glucose level, the PHQ9, and the Duke Health Profile. Other services include diabetes education, nutrition counseling, counseling, health literacy, and preventive care education. The data collection methods are described in detail in our earlier publication (1). Information was collected on all participants who presented for care on the mobile medical van (UniMóvil); there were no exclusion criteria.

The community comprises families of Mexican descent who have poor healthcare access, low socioeconomic reserve, and poor transportation resources. The participants represented the general population of the Colonias of Cameron and Hidalgo counties. The Area Deprivation Index (ADI), created to share measures of neighborhood disadvantage for research, program planning, and policy development, ranks the region as the most disadvantaged block groups (33) and is significantly impacted by social determinants of health, health disparities, and poor healthcare access.

## Statistical analysis

This report further analyzes the convenience sample of 894 patients evaluated on UniMovil between 2016 and 2018 using anonymized linked healthcare data. We calculated a Frailty Index described in an earlier publication (1). Following the literature, a dichotomous frailty variable, denoted as dFI, was constructed by scoring subjects with a frailty index of 0.25 or greater as 1 and 0 (20–22). Logistic regression analysis (yielding odds ratios) was used to determine associations between 19 possible variables and frailty. Backward stepwise model selection was used to determine the best models. Receiver operating characteristic (ROC) curve analysis was performed to compare the relative performance of the finalized models to classify frailty. All statistical analysis was conducted in the R statistical package version 4.2.0.

## Results

**Figure 1:**
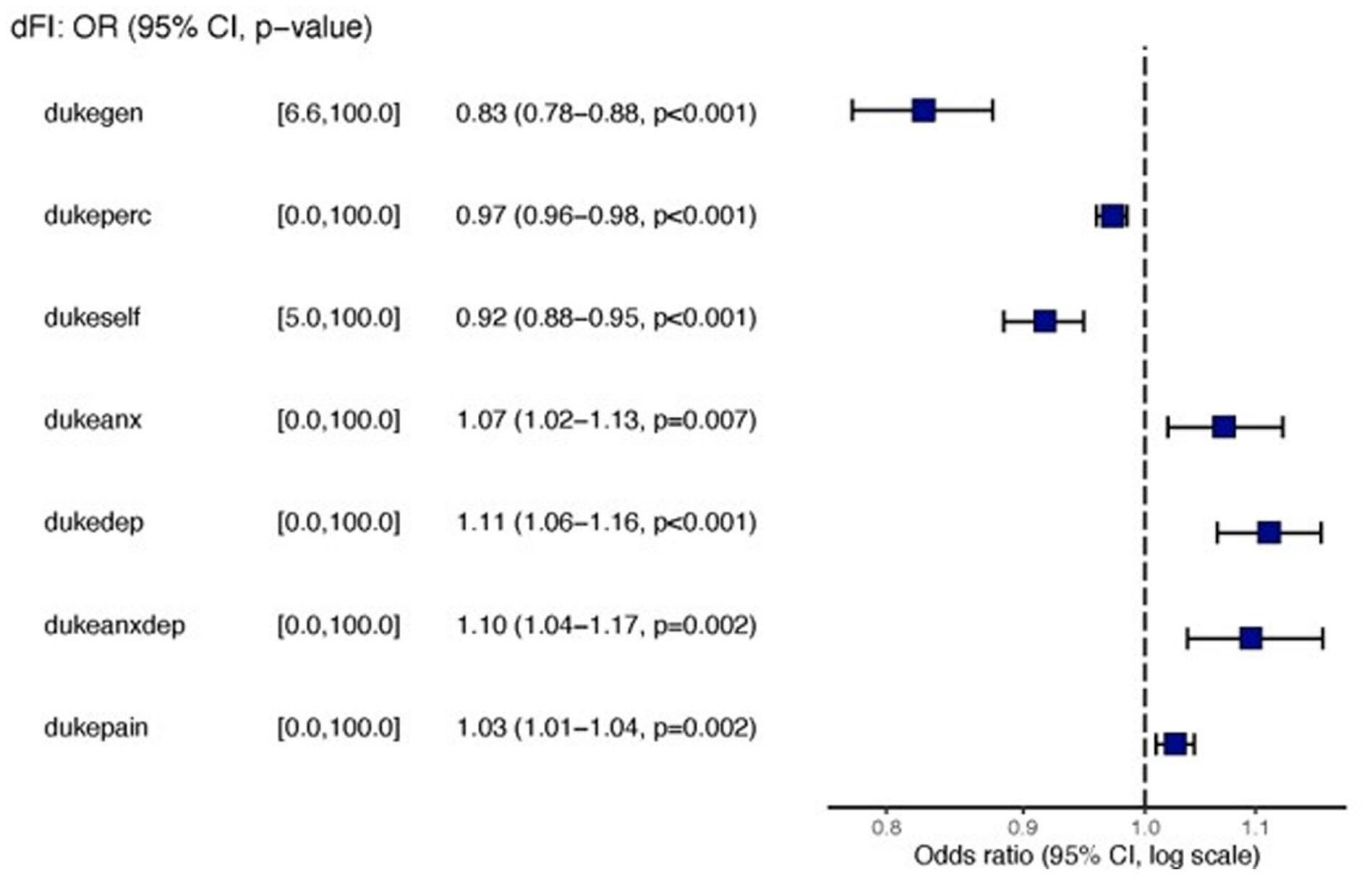
Forest plots for Model 1 with general, perceived, self-esteem, anxiety/depression and pain as independent predictors.

**Figure 2:**
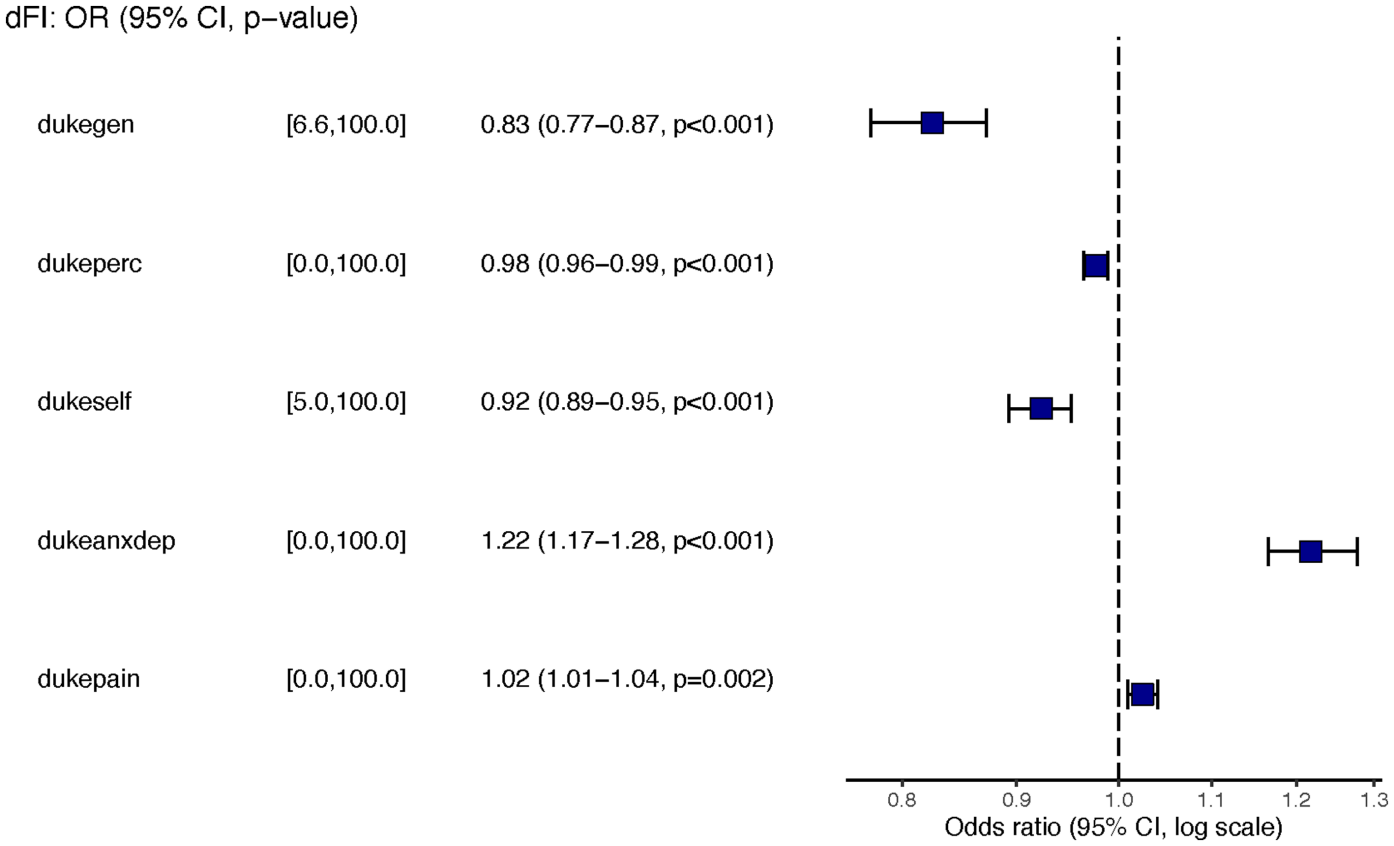
Forest plots for Model 2 with General, perceived health, self-esteem, anxiety/depression, and pain as independent predictors.

**Figure 3:**
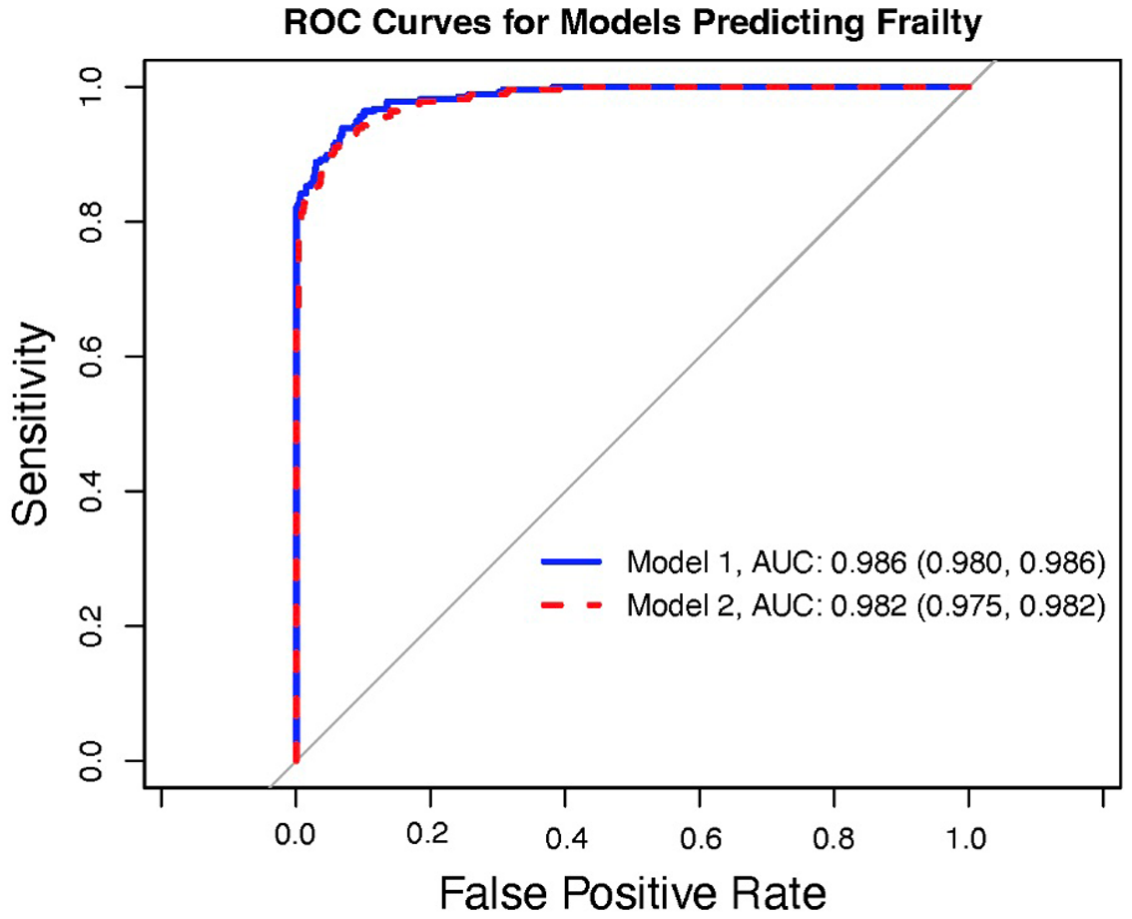
ROC curves for Models 1 (solid blue line) and 2 (dashed red line) with AUC for both.

The total and sex-specific prevalence for clinical outcomes and the Duke Health Profile by total and sex-specific samples are shown in Tables 1 2. Model selection identified a model, Model 1, with the Duke Health Profile variables General, Self-esteem, Perceived, Anxiety, Depression, Anxiety-Depression, and Pain as significant independent predictors of dFI (Figure 1). However, because Anxiety, Depression, and Anxiety-Depression are highly intercorrelated, we formulated a reduced model, denoted as Model 2, where the predictors are the same as Model 1 but now exclude Anxiety and Depression (Figure 2). Figure 3 presents the ROC curves of the two models, which shows that they are statistically indistinguishable.

**Table 1:** Total and sex-specific prevalence for clinical outcomes.

Trait	Males (*N* = 272)		Females (*N* = 622)		Value of p^*^
	Prevalence/S.D.		Prevalence/S.D.		
Normal weight	17	0.02	14	0.01	0.16
Overweight BMI 26–29	31	0.03	29	0.02	0.34
Obese BMI > 30	53	0.03	57	0.02	0.16
Normal HbA1c < 5.5	39	0.03	37	0.02	0.28
Pre-DM HbA1c 5.6–6.5	29	0.03	31	0.02	0.28
DM HbA1c > 6.5	32	0.03	33	0.02	0.49
HTN > 140/90	46	0.03	36	0.02	0.00
Cholesterol ≥200	7	0.02	6	0.01	0.19
Triglycerides ≥200 mg/DL	60	0.03	48	0.02	0.00
Low HDL-C ≤ 40 mg/DL	7	0.02	6	0.01	0.19
PHQ9 ≥ 10	17	0.02	19	0.02	0.23

*Prevalence differences were tested using a difference of proportion Z-statistic that is normally distributed for a one-tailed test. S.D., Standard Deviation.

**Table 2:** Total and sex-specific scores for HrQoL.

Domain	Males	Mean	S.D.	Females	Mean	S.D.	Value of p^*^
	*N*			*N*			
Physical health	272	65.9	22.5	622	60.9	21.55	0.01
Mental health	272	77.0	19.2	622	71.6	17.6	0.00
Social health	272	67.9	17.0	622	68.8	17.4	0.06
General health	272	70.1	15.6	622	67.0	14.3	0.00
Self-perceived health	272	71.8	27.3	622	65.7	28.6	0.00
Self-esteem	272	78.9	16.0	622	78.3	16.0	0.33
Anxiety	272	31.6	18.6	622	33.8	16.5	0.04
Depression	272	29.8	21.0	622	34.8	19.0	0.00
Anxiety-depression	272	26.8	19.2	622	31.2	17.2	0.00
Pain score	272	43.0	28.6	622	48.4	29.9	0.01
Disability	272	9.7	21.3	622	8.03	20.3	0.32

Unpaired sample *t*-test for one-tailed hypothesis. The first 6 (red) domains measure function, with high scores indicating good health. The second 5 (blue) domains measure dysfunction, with high scores indicating dysfunction.

Similar to the findings of other researchers (34), we determined that frailty was more common in young women than men but increased exponentially as males aged. After logistic regression analysis (yielding odds ratios) to determine associations between 19 possible variables and frailty, we found the associations were present only for the HrQoL variables. The prevalence of hypertension and hypertriglyceridemia was higher in men than women (Table 1). Men scored better than women in all four domains of HrQoL function (physical health, mental health, social health, general health, self-perceived health, and self-esteem). Women scored worse in anxiety, depression, anxiety-depression, and pain (Table 2). Residents of the two Colonias scored similarly on 10 of the 11 domains of the Duke Profile other than perceived health. Males scored worse on five domains that measure function (i.e., physical, mental, social, general, and perceived health). For the five domains assessed by the Duke Profile (anxiety, depression, anxiety-depression, pain, and disability), women consistently scored worse than men in all domains except for the disability domain.

## Discussion

Although we previously demonstrated that a Frailty Index that uses standard clinical and HrQoL measures is helpful in the South Texas population (1), what is novel is that the HrQoL measures, more than the biological measures of health, are the best predictors of frailty. Frailty is a state of increased vulnerability to disproportionate change in health due to the accumulation of small damages (35) and is linked to increased falls, hospitalizations, morbidity, mortality, and reduced quality of life (29). Resilience to frailty includes physical, genetic, social, and psychological moderators (2, 4, 26–28, 30, 31). Although not designed as a screen for resilience, HrQoL is moderated by social, physical, and mental health functions, and the Duke Profile HrQoL was created as a community-based screen for the same measures of the moderators of resilience (35–38). The Duke Profile HrQoL variables that best predict frailty include self-esteem, perceived health, anxiety, depression, and pain.

Whereas function (perceived health, general well-being, and self-esteem protect against frailty). Dysfunction differentially affects women, possibly due to the increased stress women living in Colonias endure (1, 39). Perception of health is also a significant predictor of frailty. Like other researchers, our findings support that psychosocial variables (depression, perceived social support, trauma account, and trauma severity) are associated with the risk of frailty (2). This is congruent with how older adults define aging (active participation, having good social and family support, thinking positively, active living, engagement, optimism and/or positive attitude, spirituality and/or religiosity, self-efficacy and/or self-esteem, gero-transcendence, spiritual well-being, and engagement with life) (26, 27, 31). The psychosocial variables, if present, increase resilience (28, 30, 40–43). Resilience is achieving, retaining, or regaining physical or emotional health after illness or loss (44). Our findings may reflect the associations between cultural stressors (acculturation and discrimination) and cultural values (family, respect, ethnic identity, and perseverance) that play a role in overcoming adversity and suggest that although biological determinants of health are essential to frailty development, HrQoL factors supporting resilience are as crucial for preventing frailty (45, 46).

### Implications for research

We know that economic distress in Mexican American neighborhoods is strongly associated with the odds of being frail (47). Elderly immigrant populations affected by co-morbid chronic illness (18) and loneliness are more often frail. Interventions to improve frailty should focus on both mental and physical illness, as well as HrQoL (48, 49).

Further investigation into how the interaction between resilience and HrQoL affect frailty is needed. Chronic illness affects frailty, but if HrQoL has more predictive power, focusing on reducing mental health and social stressors that increase resilience may reduce frailty in vulnerable populations. The Health Belief Model may explain the importance of HrQoL in developing frailty. The model focuses on individual beliefs about health conditions (perception of severity, perceived susceptibility, perceived benefits, and self-efficacy), which predict individual health-related behaviors. If confidence and self-efficacy can alter behavior, these may protect against frailty through positive behaviors that support resilience, such as diet, reduction of at-risk alcohol/substance use, and sedentary lifestyle. The Resilience Activation Framework may serve as an implementation guide to mitigate adverse responses to biopsychosocial stressors (50).

Our study includes a comprehensive Frailty Index that employs validated measurement tools widely used by clinicians. The sample is a convenience sample; the data are cross-sectional, and the picture of frailty in the Colonias may not be generalizable to other populations. Further research is necessary to determine if other deficits, including cognitive decline, cancer, disease prognosis, and chronic illness complications, change the predictive value of deficits included in a FI.

## Conclusion

This study is the first to determine the relative importance of HrQoL and subsequent resilience in the development of frailty in Mexican Americans on the U.S.-Mexican border. While FI consistently predicts frailty regardless of the number and type of variables used to calculate frailty, our findings suggest that HrQoL is more critical for predicting frailty in the Mexican American population of South Texas. Our results indicate we can decrease frailty by addressing modifiable mental health and social stressors.

## Data availability statement

The datasets presented in this study can be found in online repositories. The names of the repository/repositories and accession number(s) can be found at: Manusov (32).

## Ethics statement

The studies involving humans were approved by University of Texas Rio Grande Institutional Review Board. The studies were conducted in accordance with the local legislation and institutional requirements. The participants provided their written informed consent to participate in this study.

## Author contributions

EM was the Principal Investigator on the United Health Foundation grant, collected data, analyzed data, wrote, and was substantially involved in editing the manuscript. VD analyzed the data, completed the statistical analysis, and was substantially involved in editing. SW-B was substantially involved in the development of the concept, manuscript development, and editing. All authors contributed to the article and approved the submitted version.
